# Trichome formation in *Nicotiana benthamiana* is induced by certain *Agrobacterium tumefaciens* strains

**DOI:** 10.3389/fpls.2026.1762747

**Published:** 2026-04-28

**Authors:** Jian Chen, Phil Hands, Manish Patel, Leilei Yang, Chengcheng Zhong, Neil Smith, Ming Luo, Michael Ayliffe

**Affiliations:** CSIRO Agriculture and Food, Canberra, ACT, Australia

**Keywords:** *Agrobacterium*, cytokinin, development, transient, trichome

## Abstract

Plant trichomes are specialized epidermal appendages found on various organs of many plant species. They protect against abiotic and biotic stresses, and agricultural, medicinal, and industrial products are derived from them. Here, we show that infiltration of *Nicotiana benthamiana* leaf tissue with certain commonly used laboratory nopaline-type *Agrobacterium tumefaciens* strains induces a greater than six-fold increase, localized formation of glandular trichomes 15 days post-infiltration. This effect is *Agrobacterium* strain-specific and is caused by a *trans-zeatin synthase* gene, *tzs*, located on Ti plasmids of trichome-inducing strains. Transfer of *tzs* to strains incapable of trichome induction causes similar trichome production upon leaf infiltration. The *tzs* gene facilitates *Agrobacterium* production of the cytokinin zeatin, and similar trichome induction occurs when leaves are infiltrated with either zeatin or the related cytokinin 6-benzylaminopurine. This simple procedure enables dramatically increased trichome density to be compared biologically between adjacent isogenic plant tissues on the same leaf. Using this assay, increased trichome densities on *N. benthamiana* leaves are shown to reduce leaf predation by *Helicoverpa armigera* larvae. Finally, the cytokinin response produced by some, but not all, laboratory strains of *Agrobacterium* need to be considered when interpreting transient expression assay results.

## Introduction

Trichomes are important plant structures involved in a variety of plant biotic and abiotic protective processes, including temperature regulation, reduced water loss, light reflectance, seed dispersal, and insect and herbivore deterrence (Wagner et al., 2004; Wang et al., 2025). Two types of trichomes are produced: glandular and non-glandular. Non-glandular trichomes form physical barriers, while glandular trichomes are multicellular organs that can synthesize and store a variety of plant secondary metabolites, with some of these compounds of commercial significance. Examples include the antimalarial compound artemisinin extracted from *Artemisia annua* (Lee et al., 2023), cannabinoids from *Cannabis sativa* L (Tanney et al., 2021), and essential oils. Increasing trichome densities is particularly sought in species that provide compounds of interest for the pharmaceutical sector. Trichomes are also critical for fiber, with cotton fiber (*Gossypium* spp.) being the most important source of cellulose for the global textile industry (Kim and Triplett, 2001; Wang et al., 2021). These cellular appendages are therefore important for both plant survival and numerous commercial industries.

Studying trichomes can be inherently difficult as, firstly, in many species they can be sparsely distributed, making up only a fraction of leaf cells, and secondly, developmentally isogenic tissues often do not vary in their trichome densities, making highly controlled comparative analyses problematic. Trichome densities can be increased in some species by transgenesis (Jin et al., 2011; He et al., 2022; Peng et al., 2025); alternatively, species that are relatively abundant in glandular trichomes, such as *Solanum lycopersicum* (tomato), *A. annua* (sweet wormwood), and *Cucumis sativus* (cucumber), can be used as model systems (Chalvin et al., 2020). However, in both cases, the spatiotemporal production of these appendages cannot be controlled. A means to induce a high glandular trichome density adjacent to isogenic tissue with sparse trichome coverage, in a controlled fashion, would be a valuable tool to further understand the developmental pathways leading to trichome development and their ecophysiological effects in response to both abiotic and biotic stress.

*Agrobacterium* transient expression in *Nicotiana benthamiana* and other species is a commonly used, routine, and versatile plant molecular biology tool (Janssen and Gardner, 1989; Sparkes et al., 2006; Zhang et al., 2020). It enables rapid transient expression of genes for understanding gene function, protein localisation, and investigating protein–protein interactions (Krenik et al., 2015; Chen et al., 2023). A variety of different disarmed laboratory *Agrobacterium* strains are used for this procedure, and the potential differential influence of different bacterial strains on the infiltrated tissue is rarely considered in this context. Many commonly used disarmed laboratory strains (e.g., GV3101(pMP90), EHA101, EHA105, AGL1) are derived from the same progenitor strain, *Agrobacterium tumefaciens* strain C58, while other strains, such as, LBA4213 and LBA4404 are derivatives of *A. tumefaciens* strain Ach5 (De Saeger et al., 2021). However, isolates that have common genomes can differ in the modified Ti plasmids they contain (De Saeger et al., 2021). Of particular relevance for this study are strains GV3101(pMP90) and EHA105. GV3101(pMP90) contains disarmed Ti plasmid pMP90 (GenBank KY000034), which is a modified derivation of the Ti plasmid present in strain C58, i.e., pTiC58. In contrast, the Ti plasmid of strain EHA105, pTiBo542ΔT-DNA, is a modified version of wild-type Ti plasmid pTiBo542 (GenBank NC_010929), which is from a different Ti plasmid lineage (Weisberg et al., 2020).

Here, it is shown that a single gene encoded on pMP90, but not on pTiBo542Δ, is sufficient to cause a dramatic, localized increase in trichome formation at *Agrobacterium* infiltration sites on *N. benthamiana* leaves. This observation can be used to investigate the biological consequences of increased trichome density on isogenic tissues on the same leaf. *Agrobacterium* strains lacking this gene do not cause trichome formation or induce the cytokinin signaling response that leads to their formation, which has potential implications for interpreting transient gene expression results when different *Agrobacterium* strains have been used.

## Methods

### Plant propagation

*N. benthamiana* plants were grown under controlled environment conditions (23 °C with a 16 h light:8 h dark photoperiod) for 3–4 weeks in a 3:1 mixture of Scotts Premium Mix and Debco soil with 4 g/L Osmocote. Insects were controlled by hanging sticky insect traps in the plant growth environment.

### *Agrobacterium* infiltration assays

Cultures of *Agrobacterium* strains GV3101(pMP90), EHA105, C58, and C58C1 were grown by inoculating single colonies in Luria–Bertani liquid medium (10 g tryptone, 5 g yeast extract, and 10 g NaCl/L, pH 7.0) containing appropriate antibiotic selection and grown at 28 °C with vigorous shaking for 24 h. Cultures were then centrifuged at 2,500*g* for 5 min and resuspended in infiltration buffer (10 mM MES, pH 5.6, 10 mM MgCl_2_, and 150 μM acetosyringone) to an OD_600 nm_ of 0.8. N*. benthamiana* leaves were then syringe-infiltrated with *Agrobacterium* solutions using a 1 mL syringe. Generally, four to six infiltrations were undertaken on a single leaf, and replicate infiltrations were undertaken on multiple leaves from independent plants. The presence and absence of the *tzs* gene were confirmed in appropriate *Agrobacterium* cultures by PCR amplification of a 469 bp product from total DNA using primers GATCCAAATCGCACAAGAAAC and TCCGTCGATATCTTCCAAA, with results shown in **Supplementary Figure 1**. The location of primer binding sites in the *tzs* gene is shown in **Supplementary Figure 2**.

### Production of plasmid *pCSIRO-tzs*

A 4.3 kb fragment encoding the *tzs* gene (**Supplementary Figure 2**) was DNA-synthesized (Epoch Life Sciences, USA) and then PCR-amplified with a primer pair (primers 5′-CCCTTTTAAATATCCGATTATTCTAATAAACGCTCTTT–TCTCATGGCTCATGGCTCAGGCAGCTTCGCA-3′ and 5′-GATTTTGTGCCGAGCTGCCGGTCGGGGAGCTGTTGGCTG–GCTCTGATAGAGGAGACCAGAGTAACTTGG-3′) having homology to both the backbone sequence of binary vector pCSIRO and the *tzs* gene (underlined). pCSIRO was then PCR-amplified with primers 5′-GAGAAAAGAGCGTTTATTAGAATAATCGG-3′ and 5′-AGCCAGCCAACAGCTCCCCG-3′, which amplify the vector backbone without the T-DNA region or LB and RB sequences. The *tzs* gene was then inserted into the pCSIRO backbone by Gibson assembly. This plasmid, pCSIRO*-tzs*, was transformed into *Agrobacterium* strain EHA105, and transformants selected by growth on LB agar containing 25 ug/mL rifampicin and 50 ug/mL kanamycin.

### Scanning electron microscopy

Leaf discs of 8 mm diameter were taken from *Agrobacterium*-infiltrated areas, cleared overnight in 100% EtOH, and transferred to fresh EtOH. Discs were critical-point dried in an Autosamdri-815 automatic critical point drier (Tousimis Research Corporation, Rockville, USA) and mounted onto aluminum stubs with double-sided adhesive carbon tabs (ProSciTech, Townsville City, QLD, Australia). Samples were gold-coated using a MiniQS sputter coater (Quorum Technologies Ltd., East Sussex, UK) for 30 s under an argon environment and imaged using a Zeiss EVO LS 15 scanning electron microscope (Carl Zeiss Microscopy GmbH, Jena, Germany) under high vacuum using a secondary electron detector, 2 kV accelerating voltage, spot size 300. For determination of trichome densities on the adaxial leaf surface, trichomes were manually counted on SEM images of four *Agrobacterium* infiltration sites per strain after 6 dpi, 9 dpi, and 12 dpi, and values normalized to trichome counts/mm^2^. Trichomes could not be counted at 15 dpi due to the high trichome densities at GV3101(pMP90) and C58 infiltration sites. The 12 dpi calculations are likely an underestimate of final trichome density, as more trichome growth was apparent at 15 dpi.

### Propagation of *Helicoverpa armigera* and feeding assays

*N. benthamiana* was infiltrated with *Agrobacterium* strains of choice, and 10 dpi infiltrated leaf tissue was excised with a razor blade. Tissues were placed onto 1% agarose (Biobasic, Canada) plates, with each quadrant containing tissue infiltrated with a single *Agrobacterium* strain. Plates were then photographed, and five *H. armigera* larvae at the second instar stage were then added to each plate. A laboratory colony of *H. armigera conferta*, maintained since the mid-1980s, was used for infiltrated leaf feeding trials. Insects were reared at 25 ± 1 °C, 50 ± 10% relative humidity, under a 14 h light:10 h dark photoperiod to simulate natural conditions. Newly hatched neonates were fed an artificial diet until the second instar stage before being transferred to plates containing *Agrobacterium-*infiltrated leaf segments. Plates were then rephotographed after 12 h, 18 h, 24 h, and 36 h of insect feeding.

The extent of leaf predation was quantified via image analysis using the FIJI image processing package (version 1.53c; Schindelin et al., 2012). Briefly, the region containing each quadrant was manually isolated, and a consistent color threshold applied to isolate only green-colored pixels. For each quadrant region, the leaf area corresponding to green pixels was quantified and normalized to the area of the entire plate. For each time point, values were normalized against the area of leaf at the zero time point to determine the percentage of green tissue remaining.

Five replicate plates were assayed, and data from the zero time point and 24 h time point were compared, except for one plate image, which was selected after 36 h due to slower insect feeding (**Supplementary Figure 3**). The percentage of green tissue remaining was averaged for tissue infiltrated with the same *Agrobacterium* strain, and data compared by ANOVA with *post hoc* Tukey (https://astatsa.com/OneWay_Anova_with_TukeyHSD). For pooled data, samples were tested prior to pooling for variance heterogeneity using online calculators for the Levene (https://www.statskingdom.com/230var_levenes.html) and Bartlett tests (https://stattrek.com/anova/homogeneity/bartletts-test), respectively.

## Results

**Figure 1:**
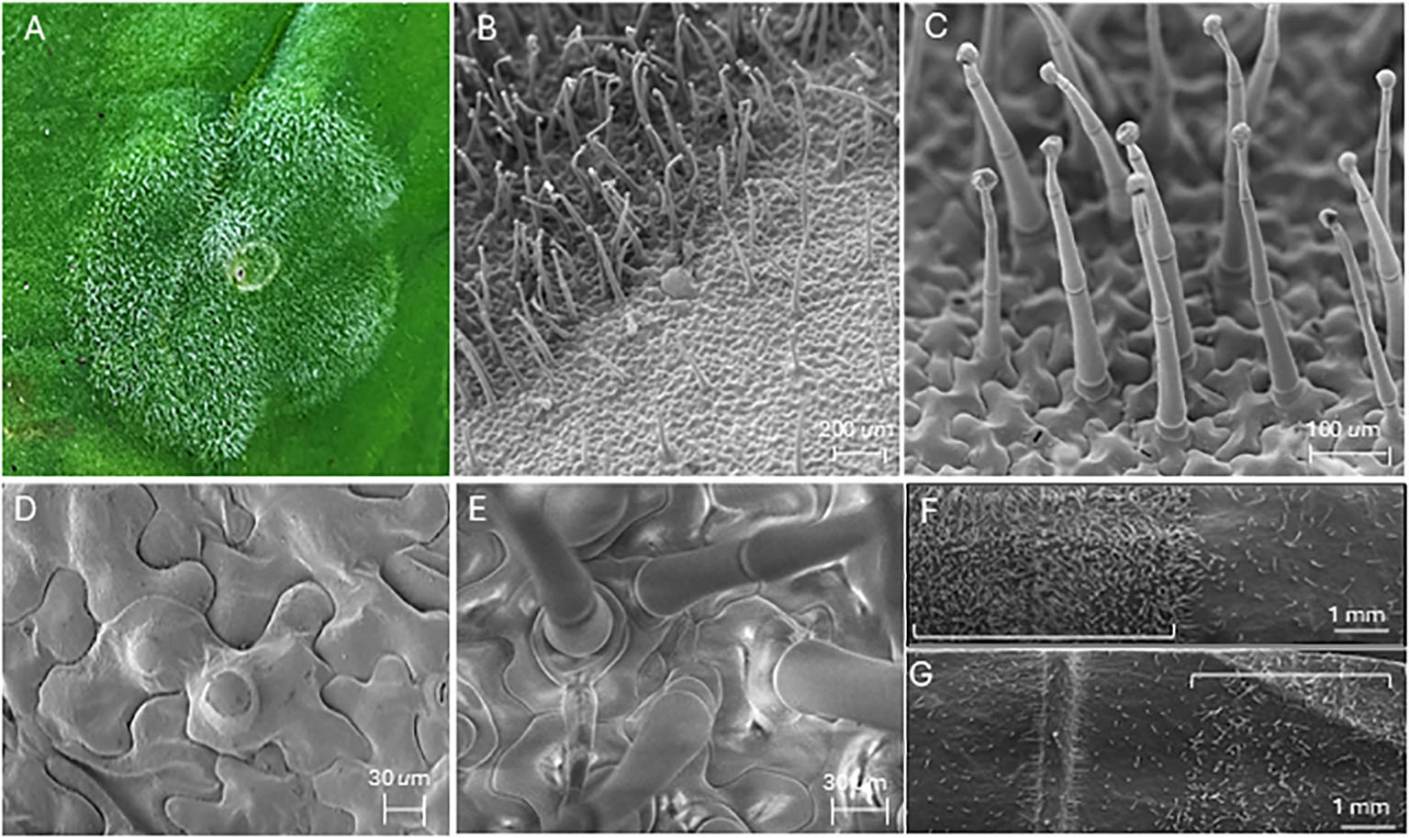
Induction of trichome growth by *Agrobacterium* infiltration. **(A)** The adaxial surface of an *N. benthamiana* leaf showing abundant, visible trichome formation at 15 dpi with *Agrobacterium* strain GV3101(pMP90). **(B)** SEM image of a GV3101(pMP90) infiltration site boundary zone on the adaxial leaf surface, with high trichome density in the infiltration site at left and reduced trichome density in adjacent uninfiltrated tissue at right. **(C)** Multicellular, capitate glandular trichomes are produced after GV3101(pMP90) infiltration. **(D)** Two emergent trichomes produced from a single epidermal pavement cell. **(E)** Multiple mature trichomes formed from single epidermal pavement cells at 15 dpi with *Agrobacterium* strain GV3101(pMP90). **(F)** Trichome densities on the adaxial *N*. *benthamiana* leaf surface following infiltration with *Agrobacterium* strain GV3101(pMP90). The infiltration zone is marked with a bracket (at left), with adjacent uninfiltrated tissue at right. **(G)** Trichome densities on the abaxial *N*. *benthamiana* leaf surface following infiltration with *Agrobacterium* strain GV3101(pMP90). The infiltration zone is marked with a bracket (at right), with adjacent uninfiltrated tissue at left. Images in **(F, G)** were assembled from multiple SEM images stitched using the ImageJ plugin MosaicJ (Thévenaz and Unser, 2007).

*N. benthamiana* leaf tissue (abaxial side) was infiltrated with *A. tumefaciens* strain GV3101(pMP90). Three leaves on each of four plants were infiltrated with four sites per leaf. At 14 dpi, abundant adaxial trichome formation occurred at infiltration sites that was absent in adjacent uninfiltrated tissue, both visibly and by scanning electron microscopy (SEM) (**Figures 1A, B**; **Supplementary Figure 4**). Infiltrations were repeated, and the vast majority of trichomes at infiltration sites were multicellular, capitate glandular trichomes (**Figure 1C**), previously described on abaxial and adaxial leaf surfaces of *N. benthamiana* (Slocombe et al., 2008). At 6 dpi, emergent trichomes were observed on epidermal pavement cells, with multiple trichomes on some cells that matured at 12 dpi–15 dpi (**Figures 1D, E**). An increase in abaxial trichome density was also observed at infiltration sites, although not as dense as on the adaxial surface (**Figures 1F, G**). In contrast, no evidence of trichome induction was observed on *N. tabacum* plants after similar infiltration with strain GV3101(pMP90) (not shown).

**Figure 2:**
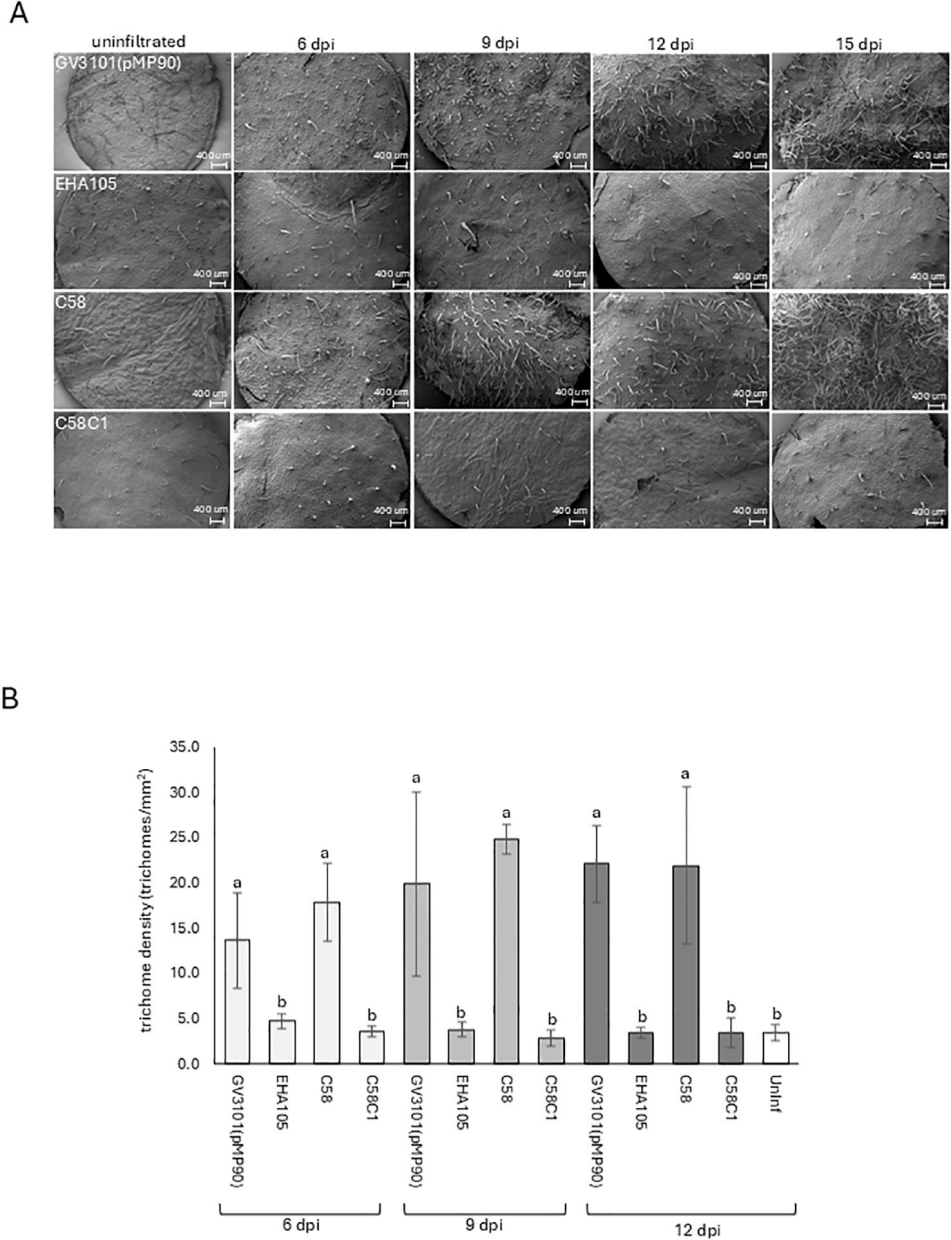
Trichome induction depends upon the *Agrobacterium* strain used. **(A)** Scanning electron microscopy (SEM) time course of the adaxial surface of *N. benthamiana* leaves following infiltration with *Agrobacterium* strains GV3101(pMP90), EHA105, C58, and C58C1. The number of days post *Agrobacterium* infiltration are indicated at the top of each column of panels. **(B)** Average trichome densities on the adaxial leaf surface at infiltration sites after infiltration with *Agrobacterium* strains GV3101(pMP90), EHA105, C58, and C58C1. Each data point is the average of four independent infiltration sites that were examined under SEM, with standard deviations shown. Statistical analyses (ANOVA with *post hoc* Tukey, p <0.05) were undertaken on data from 6 dpi, 9 dpi, and 12 dpi, respectively. Common letters above columns indicate that the data were not significantly different.

Experiments were repeated using strain GV3101(pMP90) and strains EHA105 (pTiBo542Δ), C58, and C58C1. These latter two *Agrobacterium* strains carry wild-type Ti plasmid pTiC58 (GenBank NZ_KY000040) and no Ti plasmid, respectively. After leaf infiltration, a 6 dpi, 9 dpi, 12 dpi, and 15 dpi SEM time course was undertaken. At 6 dpi, numerous small emergent trichomes were observed at GV3101(pMP90) and C58 infiltration sites in juxtaposition to larger pre-existing mature trichomes that were also present on uninfiltrated control tissue (**Figure 2A**). Over time, more emergent trichomes were observed that continued to grow, forming a dense patch at 15 dpi. In contrast, EHA105 and C58C1 infiltration did not induce trichome formation, with only sparse pre-existing mature trichomes observed (**Figure 2A**). Trichome density was six times greater on GV3101(pMP90) and C58 infiltrated sites at 12 dpi compared with other sites (**Figure 2B**).

Trichome induction by strain C58, but not C58C1, which is a C58 derivative cured of the wild-type Ti plasmid pTiC58, suggests that Ti plasmid-encoded gene(s) cause the induction. Strains GV3101(pMP90) and EHA105, which are both derivatives of strain C58, carry different disarmed Ti plasmids, further suggesting that Ti plasmid gene(s) induce trichome formation. Cytokinins can induce trichome formation when applied to plant leaf tissue (Maes and Goossens, 2010). *Agrobacterium* produces cytokinin in two ways. Firstly, by transfer of the T-DNA-encoded *isopentenyl transferase* (*ipt*) gene into the plant host (Barry et al., 1984). However, *ipt* is absent from pMP90 due to disarmament, precluding this gene from being causative. The second cytokinin source is direct bacterial production of trans-zeatin by a *trans-zeatin synthase* (*tzs*) gene located in the Ti plasmid *vir* region, which is not transferred to the plant host during infection (Beaty et al., 1986; Hwang et al., 2010). pTiC58 and pMP90 encode identical 732 bp *tzs* ORFs at nucleotides 179993–179262 and 127756–127025, respectively (GenBank NZ_KY000040 and KY000034). No *tzs* gene is encoded by pTiBo542, although the pTiC58 tzs protein and pTiBo542 ipt protein show 53% identity (Beaty et al., 1986). The presence of the *tzs* gene was confirmed in the *Agrobacterium* strains GV3101(pMP90) and C58 used in this study by PCR amplification, as was the absence of this gene in strains C58C1 and EHA105 (**Supplementary Figure 1**). Previously, GV3101(pMP90) was shown to induce shoot regeneration in tissue culture in the absence of cytokinin, whereas EHA105 could not (Han et al., 2013), consistent with zeatin production by this strain.

**Figure 3:**
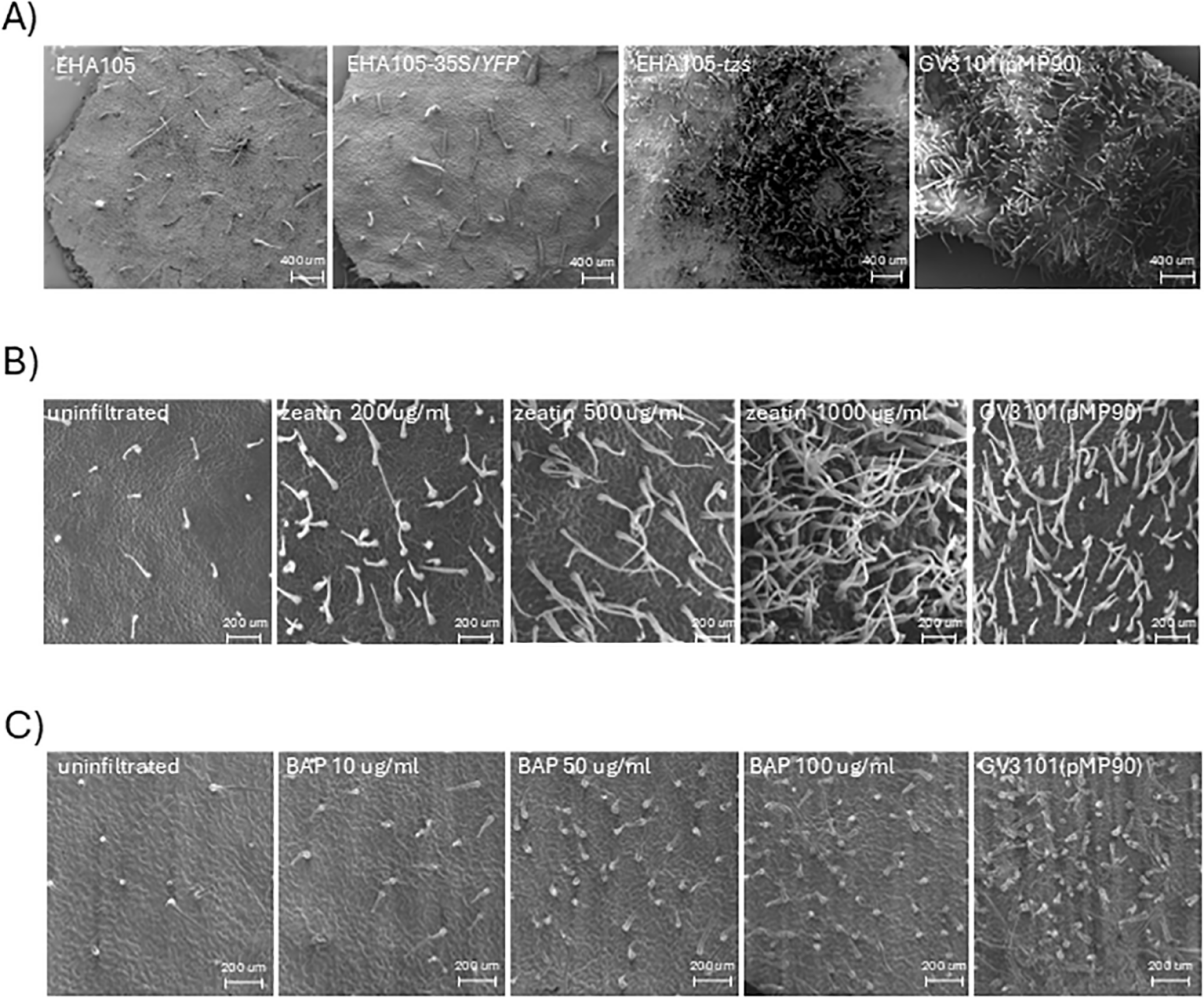
The *tzs* gene is responsible for trichome induction by *Agrobacterium*. **(A)** SEM images of adaxial leaf surfaces at 15 dpi with *Agrobacterium* strains EHA105, EHA105-*35S/YFP*, EHA105-*tzs*, and GV3101(pMP90). **(B)** Zeatin induces trichome formation in *N. benthamiana*. Leaves were infiltrated with zeatin concentrations indicated, or *Agrobacterium* strain GV3101(pMP90), and SEM images of the leaf adaxial surface at infiltration sites were taken at 21 dpi. **(C)** 6-benzylaminopurine also induces trichome formation in *N. benthamiana*. Leaves were infiltrated with 6-benzylaminopurine concentrations indicated or *Agrobacterium* strain GV3101(pMP90), and SEM images of the leaf adaxial surface at infiltration sites were taken at 15 dpi.

A 4.3 kb fragment (**Supplementary Figure 2**) encoding *tzs* (Erickson et al., 2014) was inserted into a pCSIRO binary vector that had its T-DNA border sequences removed. This construct replicates in *Agrobacterium* but is incapable of T-DNA transfer. The pCSIRO-*tzs* plasmid was introduced into EHA105 to create strain EHA105-*tzs*, which induced abundant trichome development in *N. benthamiana* upon infiltration (**Figure 3A**). The pCSIRO T-DNA binary vector backbone in an EHA105-*35S*/*YFP* control line did not induce trichome formation. The 4.3 kb fragment encoding the *tzs* gene is therefore causative for the observed trichome production. To further confirm the role of cytokinin in the observed phenotype, 200 μg/mL–1,000 μg/mL of zeatin and 10 μg/mL–100 μg/mL 6-benzylaminopurine (BAP) were separately infiltrated into *N. benthamiana* leaves, and increased trichome formation was observed at higher concentrations of both compounds (**Figures 3B, C**; **Supplementary Figure 5**). No trichome induction was observed in *N. tabacum* leaves after 10 μg/mL–100 μg/mL of BAP infiltration (not shown).

**Figure 4:**
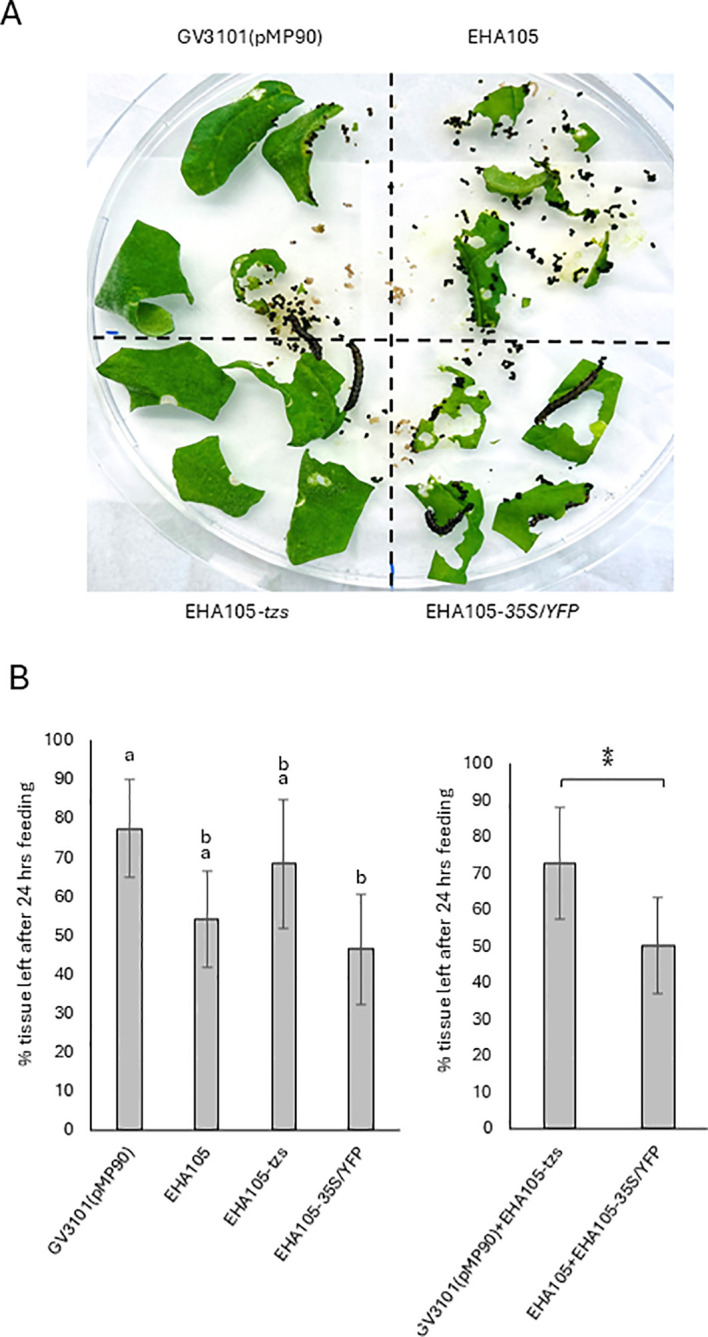
Inhibition of insect feeding of isogenic tissue varying in trichome density. **(A)** *Helicoverpa armigera* feeding after 24 h on *N. benthamiana* leaf segments previously infiltrated (at 10 dpi) with either GV3101(pMP90), EHA105, EHA105-*tzs*, or EHA105-*35S*/*YFP*. Five *H. armigera* larvae at the second instar stage were added to the plate. **(B)** Quantification of *H. armigera* feeding. The graph at left shows the percentage of infiltrated leaf tissue remaining 24 h after *H. armigera* feeding after infiltration with *Agrobacterium* strains indicated on the X-axis. Statistical analyses (ANOVA with *post hoc* Tukey, p <0.05) were undertaken and common letters above columns indicate no significant differences. The graph at right shows combined data from trichome-producing infiltrations (GV3101(pMP90) and EHA105-*tzs*) and non-trichome-inducing infiltrations (EHA105 and EHA105-*35S*/*YFP*). A highly significant difference was seen between trichome and non-trichome-inducing infiltrations (P = 0.002, Student’s t-test).

The induction of trichomes by *tzs*-expressing *Agrobacterium* enables trichome effects to be compared between isogenic tissues on the same leaf and at the same developmental stage. Insect feeding experiments were undertaken on *N. benthamiana* leaf segments infiltrated with either strain GV3101(pMP90), EHA105, EHA105-*tzs*, or EHA105-*35S/YFP*. Infiltrated leaf regions were excised at 10 dpi and placed on MS medium. Each plate quadrant contained leaf segments infiltrated with the same bacterial strain (**Figure 4A**). Five 2nd-instar *Helicoverpa armigera* (cotton bollworm) larvae were placed on each of five biological replicate plates, and the percentage of leaf tissue remaining in each quadrant was quantified after 24 h (36 h in one experiment due to delayed larval feeding) (**Supplementary Figure 3**). Tissue infiltrated with either strain GV3101(pMP90) or EHA105-*tzs* showed a reduced insect feeding trend when compared with EHA105 or EHA105-*35S*/*YFP*, although not all values were significantly different (**Figure 4B**). For subsequent statistical analysis, data were pooled for GV3101 and EHA105-*tzs* samples and EHA105 and EHA105-*35S*/*YFP* samples, respectively, as these treatments produced biologically equivalent outcomes, i.e., trichomes or no trichomes, respectively. Prior to data pooling, datasets in each pair were tested for evidence of variance heterogeneity using both Levene (Levene, 1960) and Bartlett tests (Snedecor and Cochran, 1989). No evidence of heterogeneity was observed for these comparisons (**Table 1**), providing statistical support for this pooling strategy. Comparative analysis of these two pooled datasets showed a highly significant difference in insect feeding preference, with tissue infiltrated with non-trichome-inducing *Agrobacterium* strains preferentially eaten (P = 0.002, Student’s t-test) (**Figure 4B**).

**Table 1:** Testing of variance heterogeneity among *Agrobacterium* infiltration datasets used in insect feeding assays.

Datasets compared	Levene test	Bartlett test
GV3101(pMP90) vs. EHA105-*tzs*	P = 0.75	P = 0.80
EHA105 vs. EHA105-*35S*/*YFP*	P = 0.65	P = 0.89

## Discussion

Previously, Erickson et al. (2014) reported stromule formation by plastids, changes in soluble sugar levels, starch, and delayed leaf senescence in *N. benthamiana* in response to *Agrobacterium* strain GV3101(pMP90) infiltration, but not LBA4404. Introduction of the *tzs* gene into LBA4404 enabled it to recapitulate these effects upon infiltration, as did leaf infiltration of trans-zeatin alone. LC/MS/MS measurements of trans-zeatin and trans-zeatin-9-riboside in *N. benthamiana* leaf tissue at 3 dpi showed a greater than three-fold increase in these hormones upon GV3101(pMP90) or LBA4404+*tzs* infiltration compared with buffer controls, wild-type LBA4404 or GV3101(pMP90) cured of Ti plasmid pMP90. Here, we have extended these observations to show that infiltration with *tzs-*carrying *Agrobacterium* strains also induces a dramatic increase in leaf trichome density in *N. benthamiana*. Interestingly, a similar trichome induction response was not observed in *N. tabacum*, suggesting developmental pathway differences between these two species, despite *N. tabacum* also being used as a transient *Agrobacterium* expression host.

Our study differs in that *Agrobacterium* infiltration sites were assayed >15 dpi, which enabled time for dense trichome formation to occur, although trichome induction was observed at 6 dpi. The additional post-infiltration time required for trichome development likely explains why this observation has not been previously reported, as *N. benthamiana* transient gene expression assays are usually assessed after only a few dpi. The consequences of zeatin production by *tzs*-expressing *Agrobacterium* strains are therefore profound, resulting in early subcellular responses, including changes in plastid morphology and metabolite profiles (Erickson et al., 2014), followed by a later cytokinin-induced developmental cascade leading to trichome production. Given the broad effects cytokinins have on plant homeostasis, it is likely that additional uncharacterized effects also occur at these infiltration sites.

The observation that some, but not all, commonly used laboratory *Agrobacterium* strains can cause such substantial cytokinin effects after infiltration could have significant implications for comparative interpretation of plant gene function using transient assays. Using strains not carrying *tzs* may be preferable in some instances to avoid this *Agrobacterium* cytokinin production at infiltration sites. However, in contrast, the ability to dramatically increase trichome density by this transient assay will be beneficial in further dissecting the cellular, developmental, and biological roles of trichomes, using effectively isogenic tissues. Trichomes are used as accessible models for analyzing molecular mechanisms in plant cell differentiation, cell fate, cell cycle control, and cell morphogenesis (Yang and Ye, 2013), and complex hormonal and gene regulatory network models for their development have been postulated (Wang et al., 2021). However, they are often relatively sparsely distributed in many species, and several methods have been developed to specifically enrich for them (Yerger et al., 1991; Zhang and Oppenheimer, 2004; Marks et al., 2008; Huebbers et al., 2022). Here, a six-fold increase in *N. benthamiana* trichome density is shown at 12 dpi. Past this time point, densities were too great to quantify at infiltration sites of *Agrobacterium* strains carrying *tzs*. This highly controlled induction of trichomes will enable the specific impact of trichome densities to be investigated at molecular, physiological, and biotic interaction levels. The insect feeding experiment presented here is an exemplar of the latter studies that can potentially be undertaken using this assay.

## Data Availability

The raw data supporting the conclusions of this article will be made available by the authors, without undue reservation.

## Supplementary material

Supplementary Figure 1 Identification of the *tzs* gene in *Agrobacterium* strains used in this study.

Supplementary Figure 2 Sequence of the 4.3 kb fragment encoding the *tzs* gene introduced into *Agrobacterium* strain EHA105.

Supplementary Figure 3 Replicate plates used for *Helicoverpa armigera* feeding trials.

Supplementary Figure 4 Examples of *N. benthamiana* trichome production after infiltration with *Agrobacterium* strain GV3101(pMP90).

Supplementary Figure 5 *N. benthamiana* trichome production after infiltration with zeatin.
