# Supplementary figures and images for: Trichome formation in Nicotiana benthamiana is induced by certain Agrobacterium tumefaciens strains
Source: Front Plant Sci. 2026 Apr 28;17:1762747. doi: 10.3389/fpls.2026.1762747 (PMC13161047; doi:10.3389/fpls.2026.1762747)

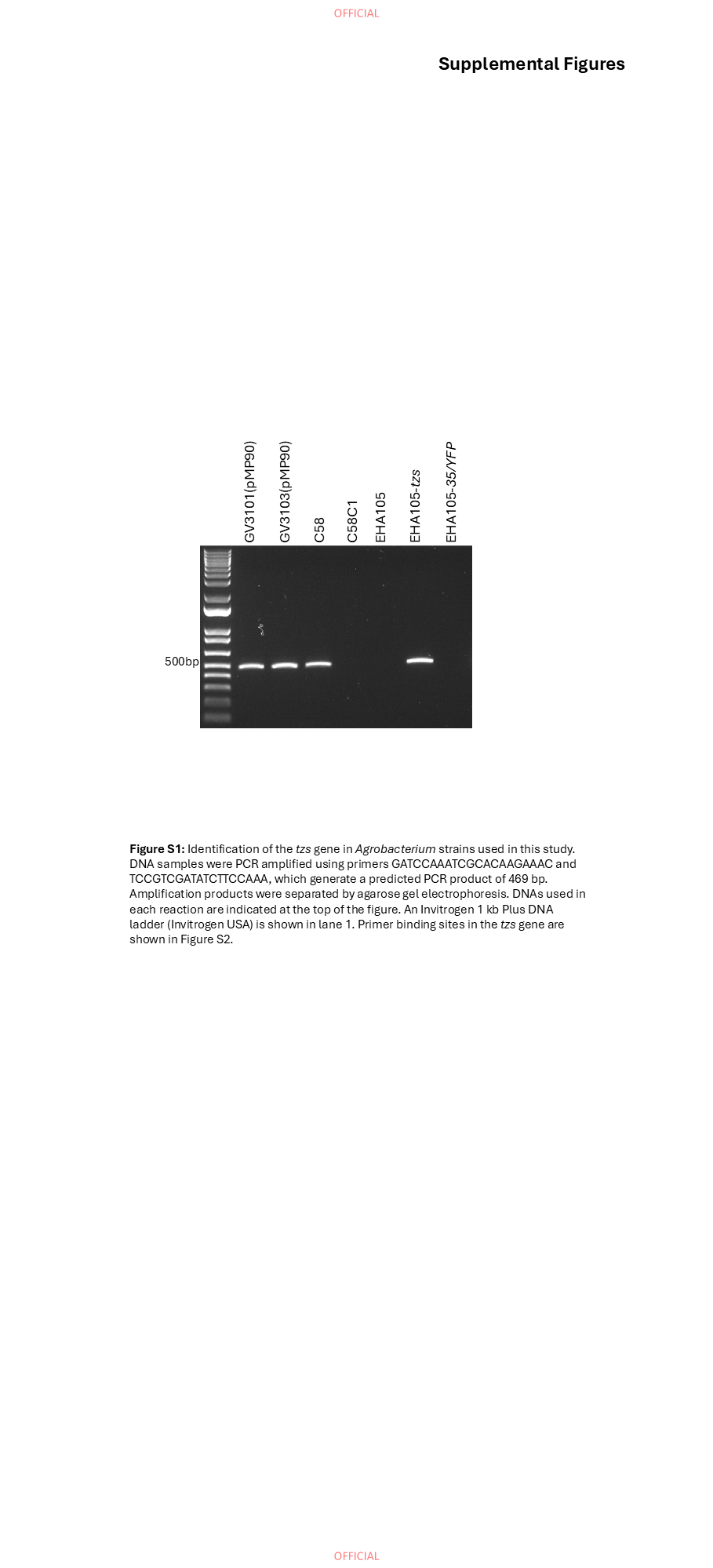

Supplement: Supplementary Figure 1 — Identification of the tzs gene in Agrobacterium strains used in this study. [file Image1.tif]

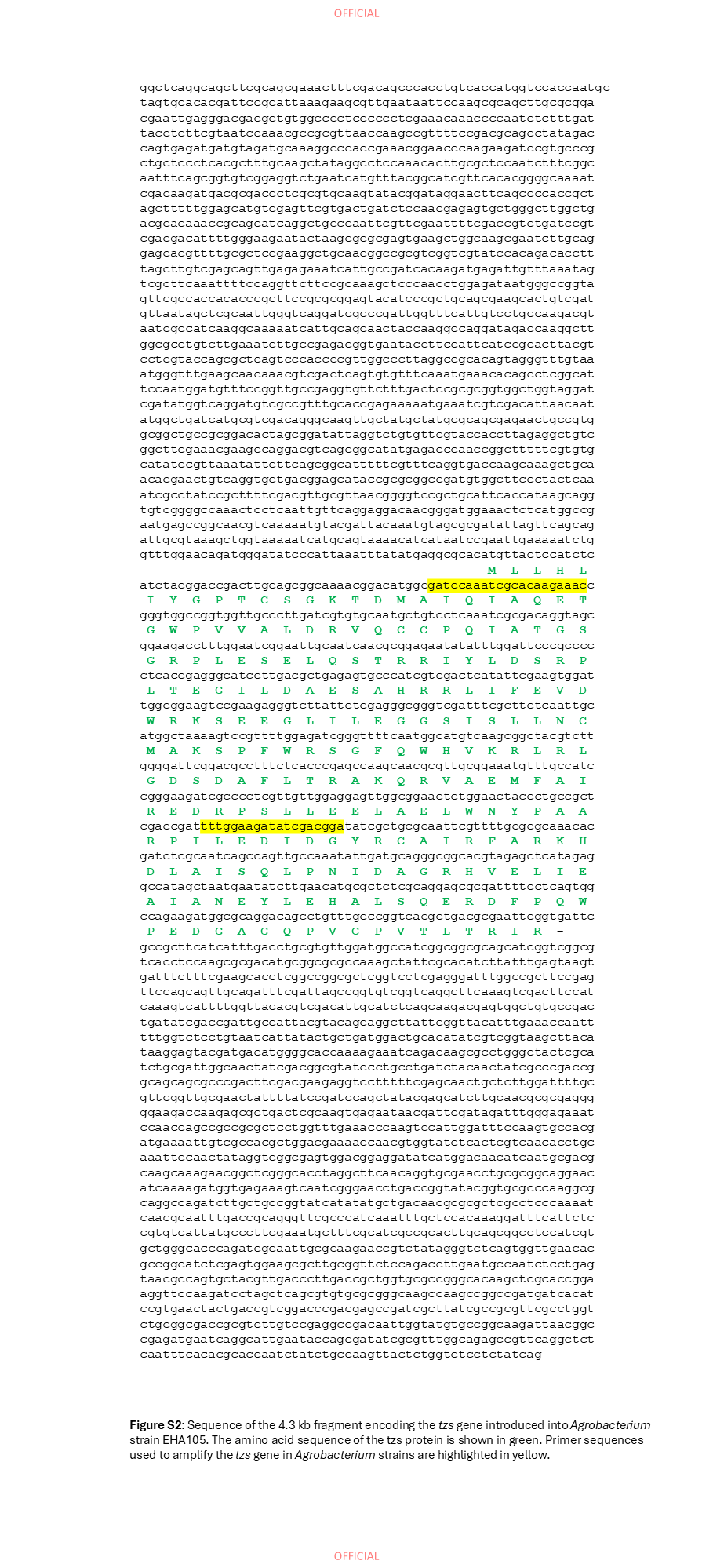

Supplement: Supplementary Figure 2 — Sequence of the 4.3 kb fragment encoding the tzs gene introduced into Agrobacterium strain EHA105. [file Image2.tif]

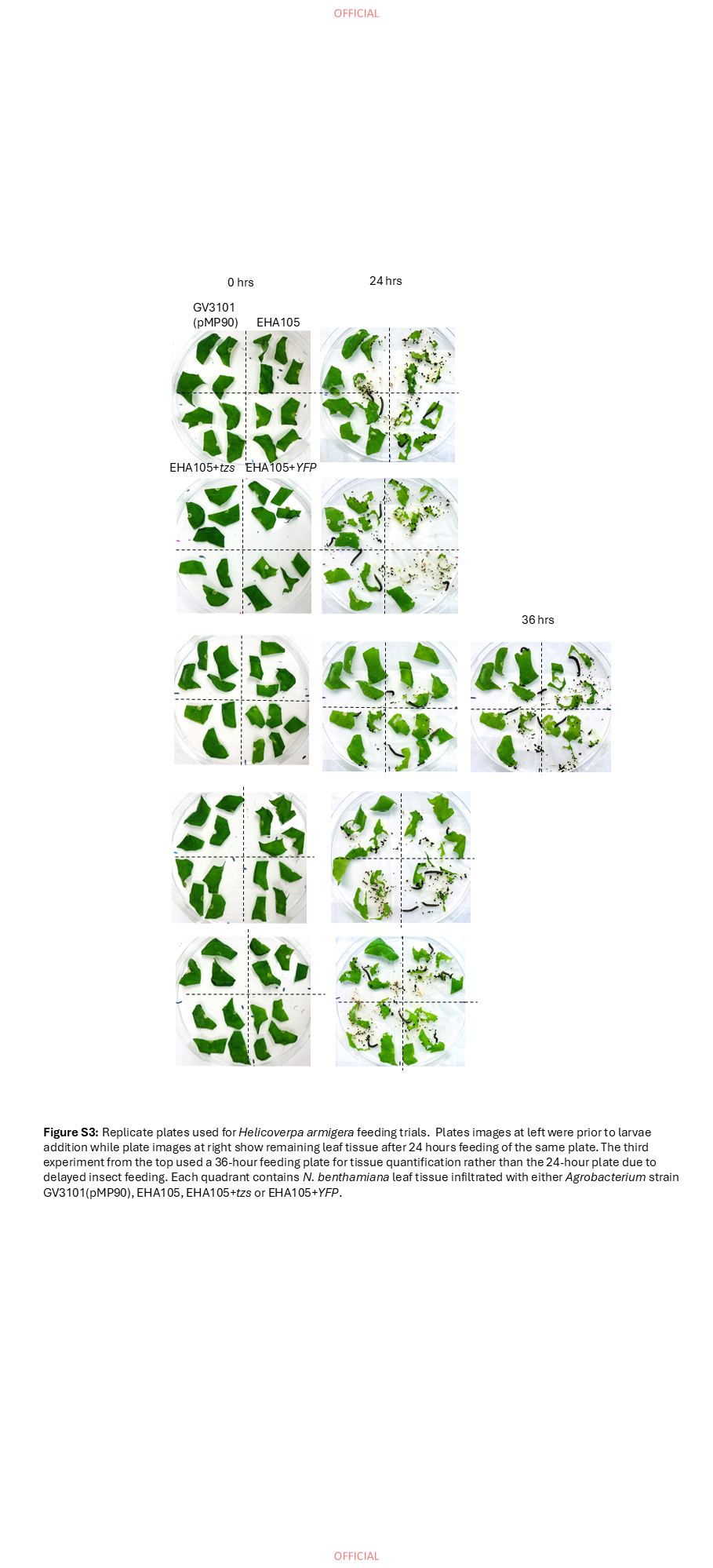

Supplement: Supplementary Figure 3 — Replicate plates used for Helicoverpa armigera feeding trials. [file Image3.tif]

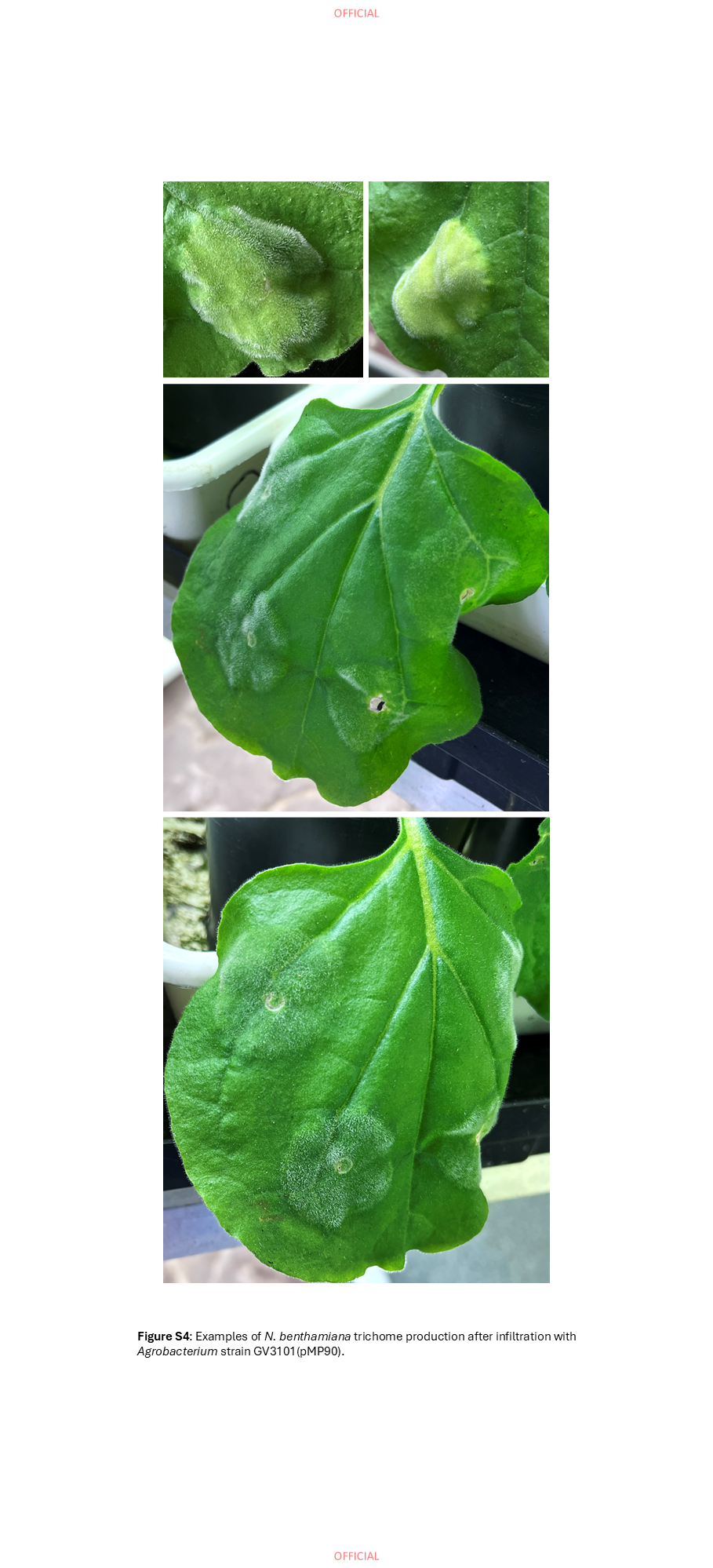

Supplement: Supplementary Figure 4 — Examples of N. benthamiana trichome production after infiltration with Agrobacterium strain GV3101(pMP90). [file Image4.tif]

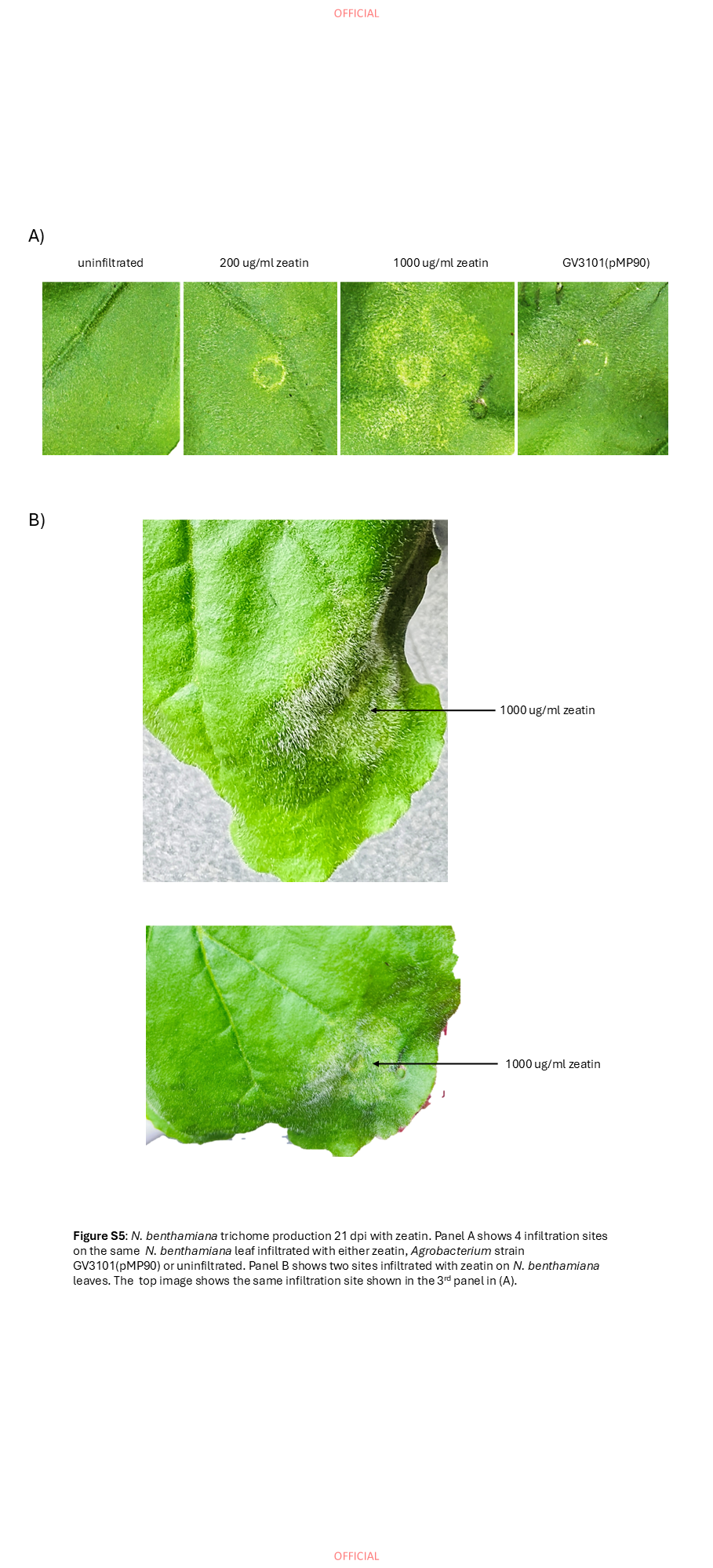

Supplement: Supplementary Figure 5 — N. benthamiana trichome production after infiltration with zeatin. [file Image5.tif]
